# Mechanisms of brief contact interventions in clinical populations: a systematic review

**DOI:** 10.1186/s12888-016-0896-4

**Published:** 2016-06-08

**Authors:** Allison Milner, Matthew J. Spittal, Nav Kapur, Katrina Witt, Jane Pirkis, Greg Carter

**Affiliations:** Work, Health and Wellbeing Unit, Centre for Population Health Research, School of Health and Social Development, Deakin University, Burwood, 3125 VIC Australia; Centre for Mental Health, Melbourne School of Population and Global Health, The University of Melbourne, Melbourne, 3010 VIC Australia; Centre for Suicide Prevention, Centre for Mental Health and Safety, Institute of Brain Behaviour and Mental Health, The University of Manchester, Manchester, UK; Centre for Brain and Mental Health Research, Faculty of Health and Medicine, University of Newcastle, Newcastle, Australia

**Keywords:** Brief contact interventions, Self-harm, Suicide, Social support, Help seeking, Postcard, Letters, Phone calls, Emergency department

## Abstract

**Background:**

Brief Contact Interventions (BCIs) have been of increasing interest to suicide prevention clinicians, researchers and policy makers. However, there has been no systematic assessment into the mechanisms underpinning BCIs. The aim of the current paper is to provide a systematic review of the proposed mechanisms underpinning BCIs across trial studies.

**Method:**

A systematic review was conducted of trials using BCIs (post-discharge telephone contacts; emergency or crisis cards; and postcard or letter contacts) for suicide or self-harm. Following PRISMA guidelines, we searched CENTRAL, MEDLINE, EMBASE, and the reference lists of all past reviews in the area. Secondary searches of reference lists were undertaken.

**Results:**

Sixteen papers provided a description of possible mechanisms which we grouped into three main areas: social support; suicide prevention literacy, and; learning alternative coping behaviours. After assessment of the studies and considering the plausibility of mechanisms, we suggest social support and improved suicide prevention literacy are the most likely mechanisms underpinning BCIs.

**Conclusion:**

Researchers need to better articulate and measure the mechanisms they believe underpin BCIs in trial studies. Understanding more about the mechanisms of BCIs’ will inform the development of future interventions for self-harm and suicide.

## Background

Brief Contact Interventions (BCIs) are low resource, non-intrusive interventions that seek to maintain long-term contact with patients, without the provision of additional therapies [1, 2]. BCIs have mostly been used with clinical populations following presentation to an emergency department (ED) for self-harm, self-injury, self-poisoning or suicide attempt. BCIs have also been applied in other populations, notably with psychiatric hospital inpatients, as a form of after-care [3] and with young people seeking outpatient mental health treatment in the community [4]. BCI interventions commonly use emergency “green” cards, phone calls, letters, postcards or text messages to keep in contact with participants. These are all similar in that they do not involve any face-to-face therapeutic contact with patients. Rather, contact is made in either verbally (over the phone) or in written form (green cards, letters and postcards). BCI letters, postcards and phone calls allow for multiple contacts with patients over time, while green cards may only have one formal contact with participants. The content of BCIs differs between studies, but generally involves a short sentence expressing concern for the patient and emphasising the availability of help should it be needed.

Recent reviews of BCIs [5, 6] and national clinical guidelines [7] highlight the potential of BCIs as a low cost intervention that could be effective in reducing repetition of self-harm. Reducing the burden of self-harm could also be beneficial to hospitals, since a proportion of self-harm cases are repeat presentations [8–10]. Further, qualitative studies with those who self-harm have identified the importance of proactive, early interventions that provide a sense of genuine care post-discharge [11]. A number of authors [3, 12, 13] suggest that BCIs possess these qualities.

Evidence regarding the effectiveness of BCIs is, at present, mixed [5]. Because of this, researchers have argued that there is a need for further evaluation of these interventions [1, 5]. Aside from continuing questions about whether or not these are effective, there has been relatively limited attention paid to the proposed mechanisms underpinning BCIs. Investigating the mechanisms of action of BCIs could provide clarity on the critical elements, including why BCIs may or may not be effective, and is thus important information for clinicians, patients and for those developing therapeutic options for self-harm.

We (AM, GC, MS, JP) have previously published a meta-analysis of BCI studies, which produced mixed results regarding the efficacy of these interventions on reducing repetition of self-harm. Drawing on this past review as a background, the aim of the current paper is to further examine these same studies to assess the mechanisms authors suggested might underpin BCIs for self-harm and suicidal behaviour outcomes (with or without explicit intent). We acknowledge that the evidence for BCI mechanisms is based on the rationale provided by the original study authors, which may have been developed following their conduct of the trial. Given this, we assessed the plausibility of these proposed mechanisms by drawing on additional evidence and research in related fields of psychology, sociology and public health.

## Method

An overview of the systematic search has been presented previously [5], but is briefly described below. The review protocol was based on the Preferred Reporting Items for Systematic Reviews and Meta-Analyses (PRISMA) (http://www.prisma-statement.org/).

We undertook a systematic search of recent systematic reviews of psychosocial post-discharge interventions for self-harm patients [2, 7, 12, 14–18]. If these studies covered the same topic as our review then these (and all cited articles within it) were eligible for inclusion in this review. We also conducted a search of the CENTRAL, MEDLINE and EMBASE electronic databases for any other studies. An example of a search strategy for CENTRAL was: (((self-harm OR suicide) AND (intervention AND post-discharge AND postcard) OR (brief contact AND follow up AND care))). No language or additional limits were applied. We also undertook a secondary search of the reference lists within all retrieved articles. We contacted authors to provide additional details needed for the review of the retrieved studies, and to provide any updates or new data on published work. The initial searches and shortlisting were undertaken by the first author (AM). Subsequent searches and checking was undertaken by three other authors (MS, JP, GC). Disagreements about whether to include a study were resolved by consensus.

Eligible studies were those of interventions employing: (1) post-discharge telephone contacts following presentation to ED or health care facility; (2) emergency or crisis cards (i.e., “Green Cards”); or (3) postcard or letter interventions [1]. We included studies that evaluated these approaches individually or in combination. We considered randomised controlled trials (RCTs), cluster randomised controlled trials (cRCTs), quasi-experimental and non-randomised trials as eligible for inclusion in this review. All studies considered for the review provided data on post-treatment suicide or self-harming behaviours, as identified during hospital or health care treatment. We considered all types of suicide and self-harming, regardless of communicated intent. Therefore, the studies may have included self-harm (with no intent to die), undetermined suicide-related behaviour (intent undetermined), and suicide attempt (with intent to die) [19].

### Establishment of main mechanism in BCI studies

All studies included in our original meta-analysis [5] were examined to establish the main mechanism proposed by study authors. Based on this review, the first author (AM) then proposed several overarching main mechanisms that encapsulated BCIs in these studies. These were reviewed by the co-authors, who each assessed the extent to which the mechanism proposed accurately reflected those in BCI studies. Thus, the process used to identify main mechanisms was iterative. Assessment of BCI mechanism was examined independently from the evaluation of the intervention itself. A range of terms were used to refer to suicidal behaviour and self-harm in BCI papers. We did not alter or change the original terms used by study authors.

## Results

We identified a total of 2416 articles from our systematic search of the databases and 15 articles from other sources [5]. After exclusions based on title and abstract, 65 full text articles were evaluated. From this, a further 45 articles were excluded because they were based on long-term face-to-face treatment (rather than brief post-discharge treatment), were trial protocol papers, or the study was not an RCT, cRCT or other eligible design. After exclusions 20 articles remained. As a number of authors published follow-up papers based on the same intervention sample, of these 20 articles, 14 were unique studies [3, 4, 13, 20–30], four were follow-up papers [31–34], and two were sub-studies from a larger trial [35, 36]. In each case, the control condition for each study was treatment as usual, which may have varied depending on the context of health care facilities and country. See Table 1 for further information on these 20 papers, including information on sample size.

**Table 1:** Papers on brief interventions included in Milner et al., 2014 review

Study	Design	Country	Outcome	Intervention (*n* = sample randomised)	Follow up	Results	Mechanisms
Beautrais et al., 2010 [20]	RCT	New Zealand	DSH	Postcards (*n* = 327)	12 mths	Non-significant reductions in overall DSH in IX compared to TAU Significant reduction in the rate of representati ons per person	**Nil - cites other external and methodological factors influencing results.** Result influenced by the overall treatment model, the level of support already available, characteristics of the treatment setting (page 58
Bertolote et al., 2010 [30]	RCT	Multiple	SA	Phone calls (*n* = 1867)	18 mths	3 sites increased SA in IX, 2 sites decreased SA.	**Social support** BIC may have reduced the sense of isolation, enhanced connectedness, and established a sense of a therapeutic alliance (page 199)
Bennewith, Stocks et al., 2002 [21]	Cluster RCT (in general practices)	UK	DSH	Letter to general practitioner (*n* = 2141)	12 mths	Non-significant increases in DSH in IX compared to TAU	**Suicide prevention literacy** Participants more willing to turn to their general practitioner for help in subsequent crises (page 6). Patients awareness of the interest shown by the general practitioner may have led them to seek help in future crises (page 7).
(Carter et al., 2005) [30]	RCT	Australia	DSP	Postcards (*n* = 722)	12 mths	Non-significant reductions in DSP in IX compared to TAU Significant reduction in repetitions of DSP	**Social support** “Patients requiring hospital treatment for deliberate self poisoning may believe that their situation is hopeless, that no one cares about them, or that they are viewed as incompetent and undeserving of care… It may be that, when combined with the postcard intervention, a service model that emphasises respect for the patient, high quality medical and psychiatric management, and follow-up arrange-ments on discharge is able to reduce the feelings of lack of social connectedness.” (page 4)
(Carter, Clover et al., 2007) [31]	RCT	Australia	DSP	Postcards (*n* = 722)	12 mths	Non-significant reductions in DSP in IX compared to TAU Significant reduction in repetitions of DSP	**Alternate behaviours** Participants learnt sustained alternative behaviours to self poisoning (p 552)
(Carter, Clover et al., 2013) [32]	RCT	Australia	DSP Suicide	Postcards (*n* = 722)	60 mths	Non-significant reductions in DSP in IX compared to TAU	**Suicide prevention literacy** it is possible that increased use of mental health out-patient services, general practitioners or community-based counselling services might have occurred in the intervention group (page 378) **Social support** Provides gesture of caring to counteracted feelings of loneliness … The intervention should be both genuine in delivery and linked to current services… The Postcards from the EDge intervention had some of these features (page 378) The postcards intervention might not be effective at all if delivered within the context of an uncaring, uncoordinated general hospital service that did not provide comprehensive psychosocial assessment (page 378). **Alternate behaviours** Learning of alternate behaviours, (p 379)
Cedereke, Monti et al., 2002 [22]	RCT	Sweden	SA Suicide	Phone calls (*n* = 216)	12 mths	No difference in SA in IX compared to TAU	**Nil - cites methodological factors influencing results.**
Chen, Ho et al., 2013 [23]	RCT	Taiwan	SA	Crisis card (*n* = 761)	6 mths	Non- significant reductions in SA in IX compared to TAU	**Nil - cites other external factors influencing results.** The effectiveness of the postcard intervention relies on the level of social support already available and the overall treatment setting. The differences in the healthcare models may have resulted in different effects (p 6)
Cotgrove, Zirinsky et al., 1995 [24]	RCT	UK	SA	Green card (*n* = 105)	12 mths	Non-significant reductions in SA in IX compared to TAU	**Social support** Providing patients with a place to escape too, provides a way to obtain help, provides a way to communicate distress, indicates that individuals are interested in their wellbeing (page 575)
Evans, Morgan et al., 1999 [25]	RCT	UK	DSH	Green card (*n* = 827)	6 mths	Non-significant increases in DSH in IX compared to TAU	**Nil - cites methodological factors influencing results.**
Evans, Evans et al., 2005 [33]	RCT	UK	DSH	Green card (*n* = 761)	12 mths	Non-significant increases in DSH in IX compared to TAU	**Nil - cites contextual factors influencing results** (including lack of skilled handling of those in crisis by junior doctors) (page 187)
Fleischmann, Bertolote et al., 2008 [13]	RCT	Multiple	Suicide	Phone calls (*n* = 1867)	18 mths	Significant reductions in suicide in IX compared to TAU	**Alternate behaviours & suicide prevention literacy** BIC increased the awareness of suicide attempters about the problems that led to the suicidal act and helped them to find ways of solving suicidal crises (page 707). **Social support** enhanced a feeling of connectedness. Also, systematic follow-up contacts gave the patient a feeling of being seen and heard by someone (page 707).
Hassanian- Moghaddam, Sarjami et al., 2011 [26]	RCT	Iran	SA self-injury (cutting)	Postcards (*n* = 2300)	12 mths	Significant reductions in SA in IX compared to TAU	**Social support** Expression of ongoing concern and the offer of contact if needed was successful in reducing subsequent suicidal ideation and suicide attempt in a population of hospital-treated self-poisoning individuals (page 315)
Hassanzadeh, Khajeddin et al., 2010 [35]	RCT	Iran	SA	Phone calls (*n* = 632)	6 mths	Non-significant increases in SA in IX compared to TAU. Process related outcomes in favour of the IX.	**Suicide prevention literacy** IX improved attitude towards seeking support from outpatient/inpatient services, relatives and friends (page 9).
Kapur, Gunnell et al., 2013 [27]	RCT	UK	SH	Letters + phone + info leaflet (*n* = 200)	12 mths	Significant increases in SH in IX compared to TAU.	**Suicide prevention literacy, methodological factors influencing results.** Presenting to hospital with repeat episodes could reflect a reduced threshold for help-seeking or improved engagement with services engendered by receipt of the intervention (page 74)
Morgan, Jones et al., 1993 [28]	RCT	UK	DSH	Green card (*n* = 212)	12 mths	Non-significant reductions in DSH in IX compared to TAU	**Suicide prevention literacy & Social support** Offer of lifelines, by opening up availability of services, in itself can be effective even though patients may not need to use it (page 112)
Motto and Bostrom 2001 [3]	RCT	USA	Suic ide	Letter (*n* = 843)	15 yr	Significant reductions in suicide in IX compared to TAU	**Social support** Being joined to something meaningful outside oneself as a stabilizing force in emotional life (page 831)
Robinson, Yuen et al., 2012 [4]	RCT	Australia	SA DSH	Postcards (*n* = 165)	18 mths	Non-significant reductions in DSH in IX compared to TAU Process related outcomes in favour of the IX.	**Cites other external and methodological factors influencing results. Suicide prevention literacy** The majority of participants found receiving the postcard to be acceptable and more than half reported using the individual sources of help messages (page 149)
Vaiva, Ducrocq et al., 2006 [29]	RCT,	France	SA Suic ide	Phone calls (*n* = 605)	13 mths	Non-significant reductions in SA compared to TAU. Process related outcomes in favour of the IX.	**Suicide prevention literacy** Telephone contact also enables the detection of people at high risk of further suicide attempts and timely referral for emergency care (page 4
Vijayakumar, Umamaheswari et al., 2011 [36]	RCT	India	SA	Phone calls (*n* = 680)	18 /12 mths	Significant decreases in SA in IX compared to TAU	**Social support, alternate behaviours & suicide prevention literacy** Provided a social support network; by increasing awareness about the problems that led to the suicidal act and hence helping in formulating an alternate coping mechanism, by enhancing a feeling of 'connectedness' and a feeling of being 'cared for' (page 247)

*Abbreviations*: *DSH* Deliberate self-harm, *SH* self-harm, *SA* Suicide attempt, *DSP* Deliberate self-poisoning, *IX* intervention condition, *TAU* control condition (treatment as usual), *eligible* participants screened as eligible, *UK* United Kingdoms, *BIC* WHO intervention “Brief Intervention and Contact. Text in bold indicates the main mechanisms discssed in the study

Of these 20 papers, 16 provided a description of the possible mechanisms underpinning results [3, 4, 13, 21, 24, 26–32, 34–36]. These were mainly found in the introduction or discussion of papers. Following the iterative process described above, we grouped these mechanisms into three main areas and defined them as follows:

1. Social support—BCIs provided participants with a sense of connectedness and the sense they were being listened to.
2. Suicide prevention literacy—BCIs improved an individual’s knowledge about suicidal behaviours or self-harm (e.g., risk and protective factors), what help is available, and how to access this help.
3. Learning alternative behaviours—BCIs involved participants learning positive and functional alternative behaviours to self-harm.

Table 1 also describes the main proposed mechanisms of BCIs (where available) for all 20 articles (provided in bold text). It should be noted that several of the articles proposed multiple possible mechanisms underpinning BCI. Several articles provided no information.

### Social support

The provision of social support was the most commonly reported mechanism (Table 1). The protective mechanisms thought to underpin social connectedness relate to perceived care and support from others, which is thought to improve coping responses to stress [37].

The idea that BCIs improved social connections or provide an additional source of social support was suggested in nine articles. This was expressed in a variety of ways. For example, Motto and Bostrom [3] suggested “being joined to something meaningful outside oneself as a stabilizing force in emotional life”, while Carter and colleagues [32] suggested that a postcard intervention may provide a “gesture of caring to counteract feelings of loneliness”. Vijayakumar and colleagues [36] described their telephone intervention as “enhancing a feeling of ‘connectedness’ and a feeling of being ‘cared for’”. While the effectiveness of the BCI was assessed independently from proposed mechanism, it was noted that all three of these papers reported that receipt of BCIs was associated with a reduction in suicidal behaviours.

Several papers also suggested that the BCIs provide a way for participants to communicate their distress and made them feel as though they are worth listening to. For example, Cotgrove and colleagues [24] described their intervention as “provid[ing] a way to obtain help…to communicate distress, [and] indicates that individuals are interested in their wellbeing”, while Fleishmann and colleagues [13] suggested that “systematic follow-up contacts gave the patient a feeling of being seen and heard by someone”. Postcard studies also typically invited participants to contact clinicians, which may have contributed to the feeling that staff were open to hearing their concerns.

### Increased suicide prevention literacy

BCIs may also lead to improved suicide prevention literacy, which we define as an individual’s knowledge about suicidal behaviours or self-harm (e.g., risk and protective factors), what help is available and/or where to find this information, and how to access this help at times of crisis. Increased suicide prevention literacy has been used to explain results of trial papers that have reported increased contact with health services for self-harm in the intervention group (albeit being non-significantly associated in some trials—see Table 1) [21, 27, 35].

The possibility that BCIs may work by increasing suicide prevention literacy was reported in eight articles (Table 1). Kapur and colleagues [27], found an increase in admissions to hospital in the intervention group compared to controls, suggesting that the BCI intervention may have potentially led to a “reduced threshold for help-seeking or improved engagement with services engendered by receipt of the intervention”. Bennewith and colleagues [21] reported that receipt of BCIs lead to a greater willingness to contact general practitioners for help in subsequent crises. Cotgrove and colleagues [24] found that none of those who used the Green Card to readmit themselves to hospital engaged in a repeat episode of self-harm, suggesting that these participants may have benefitted from the ability to access clinical services during a time of crisis afforded by the Green Card.

Studies finding reduced self-harm have also highlighted suicide prevention literacy as a possible mechanism of BCIs. A postcard study set in Tehran [26] found significant associations between an individual’s belief that the postcards were ‘helpful in the prevention of suicide’ and the proportion of self-reported suicidal ideation during the 12 month follow-up period. Specifically, those reporting no belief in the helpfulness of the postcards were almost twice as likely to report experiencing ideas of suicide compared to those with at least some belief in the helpfulness of the cards. There were also significant associations between belief in the helpfulness of these cards and suicide reattempts in this trial. An emergency card intervention also suggested that these cards can help participants improve their knowledge about where, and when, to seek help [28]. This implies that not only can BCIs inform participants about services available to them, including the possibility of hospital re-admission at times of crisis, but also reminds participants that they are welcome to contact these services if required.

Process evaluation from a number of other studies suggested that participants found BCIs to provide useful information and were associated with an increased likelihood of help seeking [4, 29, 35]. For example, Hassanzadeh et al. [35] reported that their BCI intervention increased participants’ recognition of the need to ascertain support from various sources including outpatient/inpatient services, relatives, and friends or by telephone contact. Robinson and colleagues [4] found that participants liked receiving BCIs and that they did make use of the health-promotion strategies recommended. However, no significant effect of the postcard intervention was found on attempted suicide, although participants in both the intervention and control groups improved on measures of mental health over the course of the study.

### Learning alternative coping behaviours

Participants may have also learnt more effective coping behaviours, which was reported in one trial [31, 32]. The authors of this postcard trial inferred that some alternative behaviours may have been learnt as they documented a reduction in self-poisoning readmissions and psychiatric hospitalisations over a sustained 5-year post-intervention follow-up period. Suggested examples of these alternate behaviours included improvement in coping strategies, emotion regulation, impulse control, and self-understanding.

## Discussion

We found that the most commonly reported mechanism of BCI studies related to increased social support and suicide prevention literacy. There have also been suggestions that participants may learn alternative coping behaviours when receiving BCIs, however, more work is needed to assess if and how this is connected to actual content of the intervention. Further delineation of the mechanism of action for these interventions is also required. Below we assess the plausibility of these mechanisms drawing on findings from studies in of psychology, sociology and public health, as well as qualitative and quantitative research with those who have engaged in self-harm.

### Social support 

Observational studies suggest that those with established social relationships are less likely to engage in self-harm and suicide than those who are socially isolated [38]. For example, a study on people hospitalised following a suicide attempt reported that those with greater family and peer connectedness were less likely to have re-attempted suicide after 12-months than those with less connectedness [39]. At a population level, evidence from two national samples (in the US and UK) suggested that perceived support from friends and family was associated with decreased likelihood of a lifetime suicide attempt, controlling for a variety of related predictors [40]. Studies with self-harm patients have emphasised the importance of social support post-discharge [41, 42]. The proposed protective effect of social support aligns with evidence from studies in mental health, which have reported the importance of social support in protecting against depression [37, 43] and psychosis [44]. Intervention studies have also found that peer-delivered social support “befriending” interventions have beneficial effects on depressive symptoms [37].

There are a number of ways in which social support may be protective against suicide. First, social support may have a stabilising effect to buffer against stress [3]. Hence, persons with greater social support may perceive less need for mental health services, which suggests that supportive relationships might be a substitute for or a complement to formal treatment [45]. Other researchers suggest that those with more frequent social support contact are more likely to access mental health services following a stressful life event than those with infrequent contact (i.e., social support expedites access to services), and are less likely to require specialist psychiatric services (i.e., suggesting social support plays a stress reduction role) [46].

Further, and as noted above, BCIs provide a way for participants to communicate their distress and conveys the sense that others are interested in their wellbeing. The opportunity to talk about factors that contributed to the self-harm episode is described as a important aspect of treatment in past qualitative studies [42], which supports this as being an important part of the BCI intervention. Therefore while social support is a likely mechanism, researchers in this area acknowledge that there is a need for more work into the causal mechanisms that explain why and how social support may be protective in BCI studies.

### Increase in suicide prevention literacy

A recent cross-sectional survey among adolescents who seriously considered suicide indicated that those who sought help had greater knowledge about the availability of help resources and an understanding of these could help with their problems [47]. This aligns with studies from public health which have argued that improvements in mental health literacy lead to greater awareness and knowledge about where to seek help, and greater willingness to seek help [48, 49]. It is worth noting that previous research has suggested that suicidal populations have similar levels of knowledge about mental health problems and their treatment as people in the general community [50].

BCI interventions may also change perceptions of the care process, particularly because they seek to provide support months after a person’s initial presentation to a hospital. For example, studies have shown that those who have sought help for self-harm generally have a negative opinion of hospital-based services [51] and perceive these services as unhelpful due to poor continuity of care [52]. However, other studies have shown that adolescents who engage in self-harm and were offered therapy following discharge from hospital rated it positively [53]. Thus, the offer of help following treatment for self-harm (such as a BCI) may lead to the person viewing treatment in general as more supportive. Alongside this, BCIs may be effective in modifying attitudinal barriers and stigma, making it more likely for people to contact services should they need them. These explanations align with the process evaluations discussed above, which reported that participants liked receiving BCIs and found the messages they provided helpful.

### Learning alternative coping behaviours

One group of authors speculate that BCIs may involve the learning of alternative behaviours [31, 32] and cite past research using dialectical behaviour therapy (DBT), which has been associated with a reduction in suicide attempts, lower rates of hospitalisations and ED visits, and greater adherence to treatment than cognitive behaviour therapy delivered by experts [54]. However, presently it is unclear if alternative behaviours are learnt during BCI interventions and, additionally, whether these behaviours are connected to the BCIs themselves, as few trials have measured mechanisms of change for these interventions. As we mention above, examples of possible alternate coping behaviours may include: improvement in coping strategies, emotion regulation, impulse control and self-understanding. Some of these (e.g., self-understanding or improvement in coping strategies) may go hand-in-hand with greater suicide prevention literacy, which suggests that these mechanisms could overlap or, at the very least, be mutually reinforcing.

### Limitations

There are certain caveats and limitations that need to be taken into consideration in interpreting the findings from this review. First, there may be other BCI mechanisms not covered here. To a large extent, insights of this review are reflective of the degree to which intervention researchers articulated mechanisms for BCIs. It is also necessary to acknowledge that proposed mechanisms may operate differently for different subgroups within these BCI trials. For example, several studies have documented a variation in effectiveness between those with a history of multiple episodes of self-harm and first-time attempters [20, 21, 25, 30–33]. Other studies report that BCIs may benefit women but not men for some outcomes [26, 32, 35]. We acknowledge that there may also be variations based on whether individuals had expressed intent to die (e.g., self harm versus suicidal behaviours with intent) and depending on whether they had previously been in therapy. Related to this, the proposed mechanisms may operate differently depending on the specific type of therapeutic model (e.g., DBT versus psychodynamic therapy) a person has received. However, as BCI studies have generally not been powered to look at these subgroup differences, it is difficult to assess how meaningful these effects may be in clinical practice. There may also be differences between written versus verbal forms of BCIs. For example, our original meta-analysis [5] suggested that postcard interventions may be particularly effective as a form of BCI. Last, we would acknowledge that there have been additional reviews published since the time of our original publication [5]. We did not include this because it was not in our original review.

Other limitations of this review relates to its scope and strict inclusion criteria, which meant only a relatively small number of studies were eligible for assessment. We acknowledge that the strict inclusion criteria limited the number of studies we included in this review. Last, the mechanisms that underpin letters and postcards may be different from that underpinning Green Cards. In saying this, we did find that there were similarities in the proposed mechanism across BCI studies.

#### Further research

There is clearly still more work to be done in understanding the potential mechanism of BCIs in preventing suicide and self-harm. Akin to the development of pharmacological interventions in medicine in which there is a clear understanding of the biological processes that underlie the drug’s active ingredients, we suggest that researchers in the field should a priori explicitly state proposed mechanisms and articulate the process through which they believe BCIs achieve their outcomes. For example, one hypothesised pathway might be that BCIs improve the extent to which participants feel as though they are cared for, which provides them with a sense of social support and connection that they draw on when they are experiencing problems, which in turn leads to reduced self-harm. It is possible that there are further points within this process, whereby BCIs leads to the participant seeking greater support from others, which further reduces the extent to which they self-harm. In stating these hypothesised pathways, it is also important researchers understand the extent to which BCIs may operate alongside, or complement, other therapeutic approaches. Finally, future trials should assess proposed mechanisms (such as actual and perceived social support, suicide prevention literacy, learning alternative coping behaviours) using either, or preferably both, quantitative and qualitative approaches at baseline and follow-up.

## Conclusions

Given the general interest in BCIs [5–7, 14], researchers in the area have an obligation to better articulate the potential mechanisms underpinning these interventions. Plausible explanations as to their efficacy relate to the fact that BCIs provide additional social support, greater suicide prevention literacy, and assist participants to develop alternative coping strategies. At this stage, most support is found for social support and greater suicide prevention literacy. Further research into whether these are independent or mutually reinforcing mechanisms is required in order to understand the potential efficacy of BCIs in clinical populations at risk of SH or suicide.

### Abbreviations

BCI, crief contact interventions; ED, emergency-department; SH, self harm
